# Factors influencing vitamin B6 status in domestic cats: age, disease, and body condition score

**DOI:** 10.1038/s41598-024-52367-y

**Published:** 2024-01-23

**Authors:** Vy Chu, Andrea J. Fascetti, Jennifer A. Larsen, Maria Montano, Cecilia Giulivi

**Affiliations:** 1 Department of Molecular Biosciences, University of California Davis, School of Veterinary Medicine, Davis, CA USA; 2 Medical Investigation of Neurodevelopmental Disorders (MIND) Institute UCDH, University of California Davis, Sacramento, CA USA

**Keywords:** Diagnostic markers, Ageing, Metabolism

## Abstract

Limited studies are available on vitamin B6 status in domestic cats. To this end, we evaluated glutamate–oxaloacetate transaminase (GOT) activity in hemolysates with and without pyridoxal 5′-phosphate addition in two feline populations: a cohort of 60 healthy, domestic (sexually intact and specific pathogen-free) cats maintained under strictly controlled conditions with appropriate diets housed at the Feline Nutrition and Pet Care Center, and a cohort of 57 cats randomly selected between December 2022 to January 2023 that visited the Veterinary Medicine Teaching Hospital to seek care under different circumstances. The GOT activity expressed as the ratio with and without pyridoxal 5′-phosphate addition (primary activation ratio; PAR) decreased significantly with age in the healthy cohort. The PAR values normalized to age established a cut-off for vitamin B6 deficiency in both cohorts, identifying 17 of 101 animals as vitamin B6 deficient. Using machine learning, a partition-based model (decision tree) was built to identify the most important factors that predicted vitamin B6 deficiency while using the resulting tree to make predictions for new observations. This analysis, performed with all 101 cats, revealed that the diagnosis of an infectious, chronic or acute condition (0.55) was the main contributor, followed by age (0.26), and body condition score (optimal-overweight; 0.19). Thus, our study supports that vitamin B6 supplementation may be indicated in junior to adult animals diagnosed with an infectious, chronic, or acute conditions or healthy cats with body weight ranging from optimal to overweight. In older cats, even if healthy, underweight to optimal cats appear to be at risk of vitamin B6 deficiency.

## Introduction

The term vitamin B6 refers to six interconvertible compounds that share a 2-methyl-3-hydroxypyridine structure with variable substituents at positions C4 and C5, i.e., pyridoxine (PN), pyridoxamine (PM), pyridoxal (PL), and their phosphorylated derivatives pyridoxine 5′-phosphate (PNP), pyridoxamine 5′-phosphate (PMP) and pyridoxal 5′-phosphate (PLP).

PLP is the coenzyme form of vitamin B6 that is a cofactor in > 160 different catalytic functions, including transamination. Most PLP-dependent enzymes are involved in amino acid metabolism, organic acids, glucose, sphingolipids, and fatty acids, with an essential role in the metabolism of neurotransmitters, such as dopamine, serotonin, glycine, glutamate, γ-aminobutyric acid (GABA)^1,2^.

In humans, overt vitamin B6 deficiency caused by dietary insufficiency is rare in developed countries since it is present in most foods. Consequently, B6 deficiency occurs in combination with other B vitamins or is linked to some lifestyle habits (contraceptive drugs^3^, smoking^4–7^, alcoholism^8–11^) and diseases (celiac disease^12^, diabetes^13,14^ or inflammatory conditions^15–18^). Secondary vitamin B6 deficiency may result from mutations causing defects in B6 salvage pathways^19,20^, inborn errors causing accumulation of intermediates that react with PLP, and intake of drugs that reduce the availability of PLP^21^. In addition, epidemiological human studies reported age-dependent changes in vitamin B6 status, which may reflect differences in B6 metabolism^22–25^. Low vitamin B6 intake in humans is associated with an increased risk of age-related diseases^7^ (e.g., cardiovascular disease^26–30^ and cancer^3,31,32^); however, conflicting results have been reported on B6 intake and disease risk^33,34^. Despite the wealth of studies in humans, only few reports are available on B6 status in domestic pets, including cats.

Twelve studies (8 original research articles), spanning from 1959 to 1998, have focused on the status of vitamin B6 in cats. The first three studies (late 50s to early 60s) showed that vitamin B6 deficiency in cats induces microcytic hypochromic anemia with high serum iron and the development of convulsions and kidney lesions, failure to grow, emaciation, convulsions, anemia, oxalate nephrocalcinosis, and ataxia, and if left on the diet, seizures, and death^35–37^. In vitro, isolated cerebral cortex slices from the deficient animals showed decreased formation of γ-aminobutyric acid and decreased oxygen uptake when glucose was the substrate^37^. With a gap of almost 28 years, five studies emerged spanning from 1989 to 1998. In growing kittens, vitamin B6 deficiency induced reduced body weight gain, food intake, plasma pyridoxal phosphate, and pyridoxal, hemoglobin, and hematocrit, with elevated urinary oxalate, plasma tyrosine, and plasma cystathionine^38^. Also, vitamin B6 deficiency in growing kittens resulted in abnormal histopathology, particularly active tubular degeneration and oxalate deposition^39^. Dietary protein concentrations in growing kittens (as it is in humans, mice and chickens) influenced the B6 requirements: kittens fed a 30% casein diet required 1–2 mg pyridoxine/kg diet^38^ whereas when fed a 60% casein diet, the requirement was ≥ 2 mg^40^. A novel expansion of these reports was provided by the finding that brainstem auditory evoked potentials were affected by vitamin B6 deficiency in cats, linking prolonged inter-wave intervals to slowed axonal conduction velocity secondary to defective myelination^41^. Relevant to species differences (i.e., rat vs. cats), evaluation of PLP-dependent liver tyrosine aminotransferase in cats showed little diurnal variation, no correlation with food deprivation, and a twofold increase in its activity with a high-protein diet vs. a low-protein one, reflecting the key role of this vitamin in protein metabolism. Vitamin B6 deficiency for 9 weeks decreased hepatic tyrosine aminotransferase by 64–75%^42^.

Thus, no studies reported vitamin B6 status in cats as they age; only the B6 requirement for sustaining growth in kittens^37–40^ or maintenance in adults^35,36,41,42^. In contrast, in humans, it is known that PLP concentrations vary in a sex^43,44^ and age-dependent manner^43,45–48^. As the life expectancy of domestic cats increases, the occurrence of behavioral problems seen in senior cats, such as cognitive dysfunction syndrome, is significant. While the cause of this syndrome is unclear, it has been suggested that it may result from age-related neurodegeneration and defects at the neurotransmission levels, in which vitamin B6 plays a crucial role^37,41^. Notably, a rigorous meta-analysis revealed no benefit for folate in combination with vitamin B12 and/or B6 dietary supplementation over placebo in elderly human populations with diminished cognitive function secondary to Alzheimer's disease or dementia^49^. However, the 4 studies that met the eligibility criteria indicate that earlier dietary supplementation is more effective at delaying or decreasing the severity of the cognitive decline or disease risk^44^.

However, to our knowledge, no thorough vitamin B6 status assessment in cats with age is available, and less so factors that may contribute to vitamin B6 deficiency. This knowledge gap undermines the resources pet owners and clinicians need to make informed decisions regarding dietary supplements and pet food manufacturers' formulation of senior diets.

Based on human studies, we hypothesized that a B6-dependent functional biomarker in readily accessible biological fluids such as blood decreases with age in cats. Erythrocyte PLP content is a more relevant marker of vitamin B6 status than plasma PLP content because the former serves as an intracellular enzymatic cofactor^6,50^. It is directly correlated with vitamin B6 intake^51–53^, plasma PLP^54^, PL^55,56^ and PA^52^, urinary PA excretion^52^, and degree of PLP saturation of the erythrocyte aspartate transaminase, responding within weeks to vitamin B6 depletion and repletion^52,53,57^. Furthermore, erythrocyte PLP content seems a more reliable marker under conditions and diseases associated with inflammation^54^, altered alkaline phosphatase, and low albumin^56^. However, assessing the total content of PLP does not address the binding of PLP to the apoenzymes to form a functionally active holoenzyme. The functional category includes evaluating PLP-dependent transaminase activities in tissues^42^, including red blood cells. Red blood cells are rich in the PLP-dependent glutamate–oxaloacetate transaminase (GOT)^58^, whose activity responds to changes in vitamin B6 status and in-vitro-supplied PLP^48,59^. The specific test includes assessing GOT's activity and that induced by the in vitro addition of saturating concentrations of PLP. The results are the primary activation ratio (PAR^60^) and the specific activity ratio obtained with and without PLP addition. Thus, a higher PAR reflects a lower vitamin B6 status. Advantages of evaluating this ratio include overcoming differences related to various methods and subject variability^48,61^, in addition to serving as a long-term indicator of vitamin B6 status about the life span of the erythrocytes^50^ and vitamin B6 intake^62^. Most importantly, the GOT activity test is not associated with albumin, alkaline phosphatase activity^63,64^, some immune indices^65^, and kidney function.

To this end, we evaluated the PLP-dependent GOT activity in hemolysates with and without PLP addition in two feline populations: a cohort of 60 healthy, domestic cats (sexually intact and specific pathogen-free) maintained under strictly controlled conditions with appropriate diets (Feline Nutrition and Pet Care Center, School of Veterinary Medicine, the University of California-Davis, named hereafter as Cat Colony) and a cohort of 57 cats randomly selected between December 2022 to January 2023 that were brought to the Veterinary Medicine Teaching Hospital at the University of California-Davis (VMTH) to seek care under different circumstances with available blood samples (Tables 1, 2). Our second goal was to analyze the data based on the animal's age, sex, body condition score, and clinical condition to elucidate the main factors driving vitamin B6 deficiencies in domestic cats.

**Table 1:** Clinical data on cats from the Cat Colony.

Breed	Sex	Age (y)	BCS	FCoV	Notes
DSH	F	1	4/9	−	Healthy
DSH	F	2	4/9	+	Healthy
DSH	F	2	4/9	+	Healthy
DSH	F	3	4/9	−	Healthy
DSH	F	3	4/9	+	Healthy
DSH	F	3	4/9	−	Healthy
DSH	F	5	4/9	−	Healthy
DSH	F	6	4/9	−	Healthy
DSH	F	7	4/9	−	Healthy
DSH	F	8	4/9	+	Healthy
DSH	F	11	4/9	−	Healthy
DSH	F	14	4/9	+	Healthy
DSH	M	1	4/9	−	Healthy
DSH	M	3	4/9	−	Healthy
DSH	M	14	4/9	+	Healthy
DSH	F	1	5/9	−	Healthy
DSH	F	1	5/9	−	Healthy
DSH	F	2	5/9	+	Healthy
DSH	F	3	5/9	−	Healthy
DSH	F	4	5/9	+	Healthy
DSH	F	7	5/9	−	Healthy
DSH	F	7	5/9	−	Healthy
DSH	F	7	5/9	−	Healthy
DSH	F	7	5/9	+	Healthy
DSH	F	7	5/9	−	Healthy
DSH	F	7	5/9	−	Healthy
DSH	F	8	5/9	+	Healthy
DSH	F	8	5/9	−	Healthy
DSH	F	8	5/9	−	Healthy
DSH	F	17	5/9	+	Healthy
DSH	M	1	5/9	−	Healthy
DSH	M	5	5/9	−	Healthy
DSH	M	13	5/9	+	Healthy; on diet restriction
DSH	F	2	6/9	−	Healthy
DSH	F	7	6/9	+	Healthy
DSH	F	8	6/9	−	Healthy
DSH	F	6	7/9	−	Healthy
DSH	F	11	7/9	−	Healthy
DSH	F	14	7/9	−	Healthy
DSH	F	6	8/9	−	Healthy
DSH	F	7	8/9	−	Healthy
DSH	F	14	8/9	−	Healthy; on diet restriction
DSH	F	1	NA	+	Healthy
DSH	F	1	NA	+	Healthy
DSH	F	1	NA	+	Healthy
DSH	F	2	NA	+	Healthy
DSH	F	7	NA	−	Healthy

DSH, domestic short hair; F, female; M, male; FCoV, feline coronavirus; NA, not available.

**Table 2:** Clinical data on cats from the veterinary hospital.

Breed	Sex	Age (y)	Weight (kg)	BCS	Muscle condition score	Diagnosis	Diet
DSH	FS	1	5	6/9	NA	History of polyarthritis (resolved)	NA
DSH	FS	3	3.7	5/9	NA	Ileocecocolic mass, hyperglobulinemia, eosinophilia, basophilia	Wellness chicken pate, supplemented with Hill's c/d
DSH	FS	3	5	6/9	NA	Linear GI foreign body	NA
British shorthair	FS	3	3.3	6/9	NA	Suspected liver mass, pancreatitis	NA
DSH	FS	3	4	7/9	NA	Trauma from dog attack, euthanized	NA
Persian	FS	4	2.1	3/9	Severe	Uveitis, anemia, flaccid tail, pain	Royal Canin Persian dry
DSH	FS	7	4.2	5/9	NA	Weight loss, history of feline infectious peritonitis (FIP)	Hill's Healthy Advantage Oral + dry
DSH	FS	7	6.5	7/9	NA	Atopy	Hill's w/d
DLH	FS	8	4	2/9	Severe	Chronic enteropathy, hypocobalaminemia, hypofolatemia, chronic pancreatitis, upper respiratory infection, dental disease, anemia, hyperbilirubinemia	Nine lives wet food and dry food Purina
DSH	FS	8	5.4	7/9	NA	Diabetes ketoacidosis (new DM), pancreatic adenocarcinoma, urinary tract infection, acute kidney injury with chronic kidney disease (CKD), lipidemia, murmur	Hill's c/d
DMH	FS	8	5	7/9	NA	Lung mass with pleural effusion, euthanized	NA
DSH	FS	9	3.9	5/9	Normal	Inflammatory airway disease	Hill's i/d wet
DLH	FS	12	2.7	1/9	Severe	Acute B cell leukemia, possible heart disease	Purina sensitive stomach
Persian	FS	12	3	2/9	Severe	Restrictive cardiomyopathy, heart failure, CKD	Unspecified Purina Pro Plan
DLH	FS	12	3.5	3/9	NA	Laryngeal plasmacytoma, lymphoma (on chemotherapy and radiation)	NA
DSH	FS	12	4.3	6/9	Normal	Severe rhinitis, anemia, dental disease	Purina HA
DSH	FS	15	3.8	4/9	Mild	Ocular disease	NA
DSH	FS	15	5.6	7/9	Normal	Thymoma (starting radiation)	Unspecified Kirkland dry
DLH	FS	16	4.1	NA	NA	Lymphoma (on chemotherapy), hypocobalaminemia (supplemented)	Hill's y/d dry
DSH	M	0.25	1.4	5/9	Normal	Vascular ring anomaly (persistent right aortic arch), megaesophagus	Royal Canin kitten veterinary diet pureed with water
DLH	MN	0.33	1.4	3/9	NA	Cough, high BUN	Purina kitten seafood medley gravy and bits mostly canned
Scottish fold	MN	0.833	3.8	6/9	NA	Effusive FIP (positive response to therapy)	NA
DSH	MN	1	4.5	5/9	Normal	Dry FIP (remission), mild eosinophilia	Unspecified Blue Buffalo dry food
DSH	MN	1	4.8	6/9	Mild	Obstipation, spinal abnormalities (euthanized)	NA
DSH	MN	2	5.1	4/9	Normal	Chronic enteropathy, cough, hypertrophic cardiomyopathy (HCM)	Purina HA, Royal Canin PR
DSH	MN	3	2.6	2/9	Severe	Severe anemia, CKD, cardiac disease	Hill's k/d dry fed through e-tube
DSH	MN	3	4.7	5/9	NA	Systemic mycobacteriosis, polyneuropathy (euthanized)	NA
DSH	MN	3	6.4	6/9	Normal	Upper respiratory signs	Purina Urinary and turkey or canned tuna
Maine coon	MN	4	9.9	4/9	NA	Recurrent pyothorax (euthanized)	NA
DSH	MN	5	4.5	5/9	Normal	Idiopathic chylothorax (euthanized)	Purina OM
DMH	MN	5	5.6	6/9	NA	Urethral obstruction	Friskies seafood sensations dry
DLH	MN	6	5.4	4/9	Normal	CKD, cystic mass on pancreas	Purina Proplan sensitive stomach turkey and oatmeal ¼ cup kibble/day. 3 oz can tuna wet food/day
DSH	MN	6	5.8	6/9	NA	Stomatitis	Unknown dry and canned
DSH	MN	7	3.4	2/9	Severe	Likely CKD, lymphoma (on chemotherapy), hypocobalaminemia (supplemented)	Mostly unspecified treats
DSH	MN	7	4.8	6/9	Normal	Multiple myeloma (on chemo), CKD, HCM	Hill's i/d
DLH	MN	7	6.4	9/9	NA	Respiratory disease (improved, suspected parasitic), chronic enteropathy	Royal Canin Selected Protein PR
DSH	MN	9	4.2	4/9	Normal	Diabetes mellitus, chronic enteropathy, chronic pancreatitis, ocular disease	Royal Canin Selected Protein PR wet plus access to the dry as well as Royal Canin Satiety Support dry and Hill's Science Diet dry
DMH	MN	9	5.4	5/9	Normal	Lymphoma (on chemotherapy)	NA
DSH	MN	9	7.3	8/9	Mild	Diffuse enteropathy	NA
DMH	MN	10	5.3	5/9	Mild	Dental disease	Purina HA and Purina Hairball in a 3:1 mixture plus greenies and Temptation treats
DMH	MN	10	4.9	5/9	Normal	Healthy	Purina Hairball; Temptations chicken or salmon treats
DSH	MN	11	5.1	7/9	NA	Caudal neuropathy, 3rd degree AV block	Hill’s canned dry mixed with canned salmon to hide Prednisone
DSH	MN	12	4.9	4/9	Normal	CKD, anemia, pancreatitis, FIV	NA
Maine coon	MN	12	4.6	5/9	Normal	Lymphoma (on chemotherapy)	Royal Canin kitten/Purina kitten/Hill’s a/d
DMH	MN	12	5.2	7/9	Normal	Chronic enteropathy, dental disease	Royal Canin hydrolyzed Protein dry and Hill's d/d venison wet
NA	MN	13	3.8	4/9	NA	Vomiting and diarrhea	unknown dry and canned
DSH	MN	13	8.3	7/9	Normal	CKD, HCM, chronic pancreatitis, diabetes mellitus, inflammatory bowel disease (IBD), septic joint	Royal Canin multifunction renal/hydrolyzed protein dry
DSH	MN	14	3.7	2/9	Severe	Severe HCM with heart failure, hyperthyroidism, chronic pancreatitis, severe mixed hepatopathy	Nine Lives and Friskies wet + Meow Mix dry
Ragdoll	MN	14	4.3	3/9	Normal	CKD, chronic enteropathy	Hill's z/d dry
DSH	MN	14	4.5	3/9	NA	Vomiting	Hill's z/d wet
Rex	MN	14	4.3	NA	Normal	Lymphoma (on chemo and radiation), hypocobalaminemia and moderate hypofolatemia (B12 supplemented)	NA
DLH	MN	15	4.18	3/9	NA	Pulmonary carcinoma (on chemotherapy), CKD	Royal Canin Renal Support
DSH	MN	15	4.3	6/9	Moderate	Triaditis, hyperthyroidism, CKD, chronic enteropathy, HCM	Royal Canin multifunction hydrolyzed protein renal function dry (likes dry more than canned), tuna flavored wet
DSH	MN	17	4.9	5/9	Moderate	Metastatic GI mast cell neoplasia, chronic enteropathy, obstipation, CKD	Wet "Costco diet"

DSH, domestic short hair; DLH, domestic long hair; DMH, domestic medium hair; F, female; M, male; FS, female spayed; MN, male neutered; a/d intermittent or supplemental feeding; NA, not available.

## Results

### Vitamin B6 status in domestic research cats

**Figure 1:**
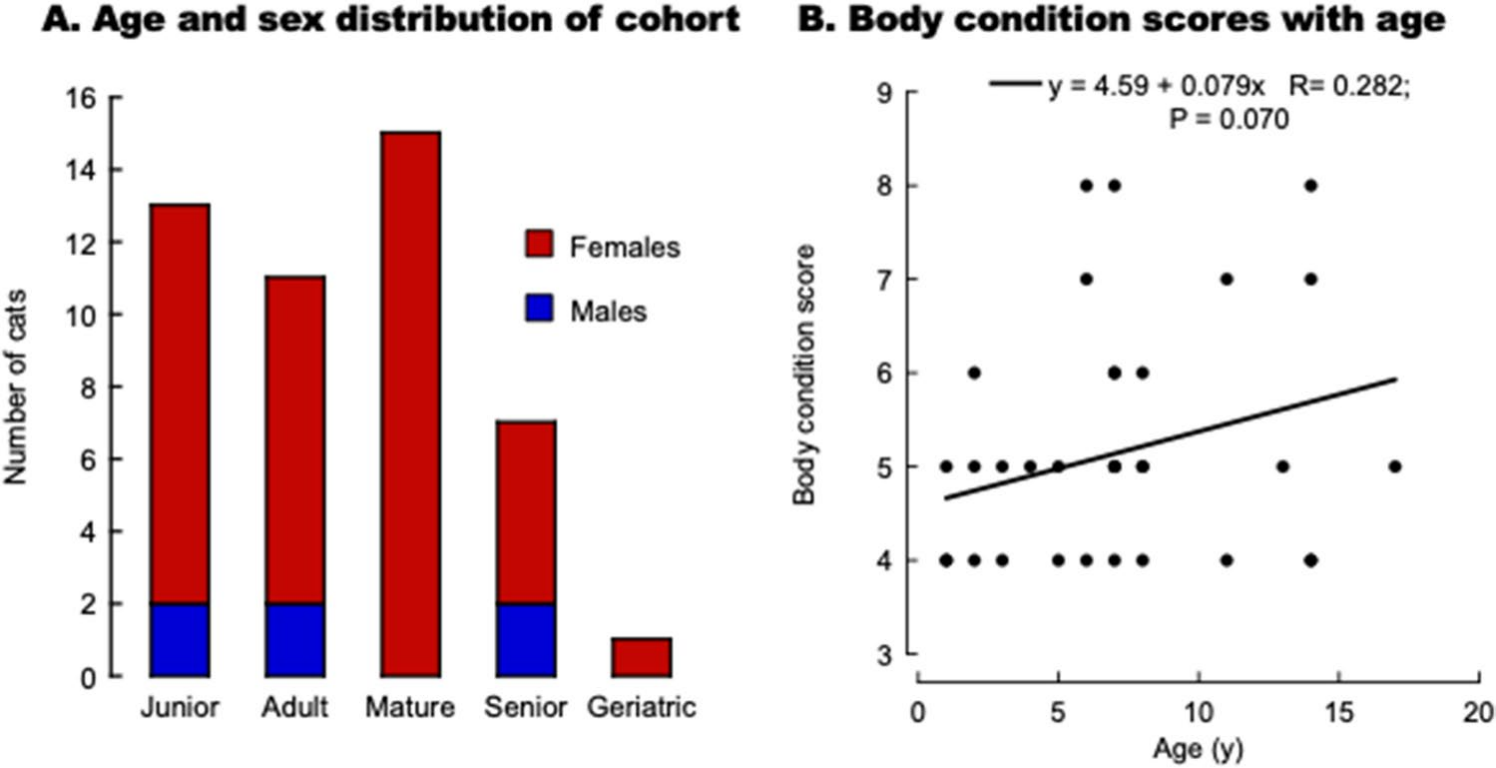
Age, sex, and body score condition distribution in domestic cats from the Feline Nutrition and PetCare Center (Cat Colony). (**A**) Age and sex distribution of the 47 domestic cats from the Cat Colony. (**B**) Association between body condition score and age in cats. The equation represents the linear fitting; P value is from Pearson's.

Blood samples were collected from 47 mostly female, specific-pathogen-free, sexually intact cats (41 females, 6 males) ranging from 1 to 17 y old (Fig. 1A; Table 1). This cohort had an almost equal representation of junior (≤ 2 y; n = 13), adults (3 to 6 y; n = 11), and mature cats (7–10 y; n = 15) with less representation of seniors (11–14 y; n = 7) and geriatric cats (≥ 15 y; n = 1). Although some cats residing at the Cat Colony were positive for feline coronavirus (tested as a part of the routine panel), they were asymptomatic and considered healthy by the standing staff and veterinarians.

To assess the overall fitness status, we used the body condition score. This parameter estimates adipose tissue, similar to the human body mass index^66^. The scale used in this study spans from 1 (emaciated) to 9 (grossly obese), with a score of 5 being optimal^66^. The average body condition of this cohort indicated that most cats had a healthy body weight (mean ± SD = 5 ± 1). For those with scores available (n = 42), the body condition score showed a positive association with age without reaching statistical significance (Fig. 1B). The higher body condition score with age may be linked to various medical conditions such as atopic dermatitis, hypertension, diabetes, asthma, ophthalmic diseases, and allergies^67^; however, all cats at the time of blood withdrawal were healthy with no signs of any of these conditions.

**Figure 2:**
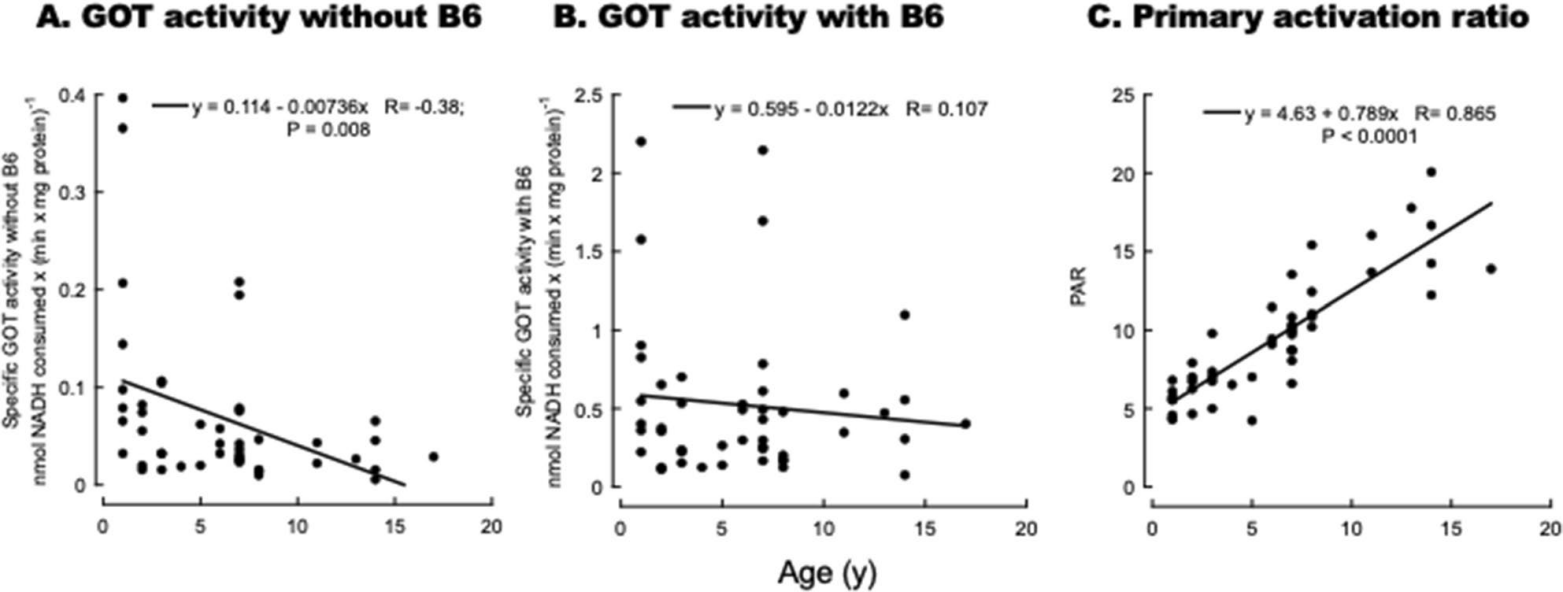
Glutamate–oxaloacetate transaminase specific activity with and without vitamin B6 addition as a function of age. GOT activity was evaluated in hemolysates as described in detail under “Materials and methods” without (**A**) and with (**B**) PLP addition. The data are presented as a function of the cat's age. Equations represent the linear fittings, and the P values are from Pearson's. The primary activation ratio (ratio of the GOT-specific activity with and without PLP addition) is shown as a function of age (**C**).

In this healthy cohort, the specific GOT activity (expressed as units/mg protein) was assessed in red blood cell samples with and without the addition of saturating PLP concentrations. First, the experimental data on GOT activities without PLP addition were analyzed by using a stepwise regression to limit regressor effect probabilities, determine the method of selecting effects, begin or stop the selection process, and run a model by including covariates age, BCS, and sex or, if using only females, BCS, and age. The minimum Bayesian Information Criterion (BIC) defined the model selection criteria as the stopping rule. The model was then run using least squares fit with significant terms (Supplementary information). As age was the only one with statistical significance when considering both sexes (P = 0.049) and marginally only with females (P = 0.109), the associations between GOT activities without PLP addition and age were fitted to a linear regression model. The GOT activity without PLP addition with both sexes declined steadily with age from 1 to 17 y (Fig. 2A), whereas upon PLP addition, the significance of the correlation with age was lost with both sexes and females only (Fig. 2B; Supplementary information).

As the degree of saturation of erythrocytic GOT by PLP is used as a status indicator of B6^68,69^, we calculated the primary activation ratio (the ratio of GOT activity with and without PLP supplementation). As indicated above, with the GOT activities, we performed an analysis based on the best stepwise model followed by the least square one. This analysis indicated that only age was statistically significant when using data from both sexes or females (P < 0.0001; Supplementary information). As such, we used a linear regression model to fit the PAR data with age. The PAR increased linearly with the age of cats (Fig. 2C; P < 0.0001), following a trend similar to that reported for humans (i.e., high in newborns and gradually decreasing throughout the lifespan^70,71^). The linear regression between PAR and age was still highly significant, with no significant slope changes even with female data only (Supplementary information). The effect of PLP addition on GOT activity in samples from older animals was between 2 and 3 times greater than on the GOT in the younger ones (10 vs. 1 y old). These results are consistent with the incubation of hemolysates with PLP from older subjects, resulting in a greater activation of GOT than in samples from younger subjects, reflecting lower vitamin B6 levels in the blood of elders^72–74^. These reports and the results presented here indicate that PLP is present in red blood cells in suboptimal concentrations for maximal GOT activity.

As described in the “Materials and methods”, the PAR values were corrected for age to establish a cut-off value for identifying vitamin B6 deficiency. Accordingly, values ≥ 1.25 of the age-adjusted mean indicated vitamin B6 deficiency. Under this condition, 6 cats (5 females, one male) were identified as having abnormally high PAR values normalized to age. The age and BCS distributions of these 6 cats indicated that the vitamin B6 deficiency was spread across ages by including two juniors (age; BCS: 1 and 2 y; 4 and 6), one adult (3 y; 5), two mature (7 and 8 y; 5 and 4), and one senior (14 y; 4) with most cats having abnormal BCS values (three underweight and one overweight). Although asymptomatic, three cats were positive for feline covid virus.

### Vitamin B6 status in domestic client-owned cats

**Figure 3:**
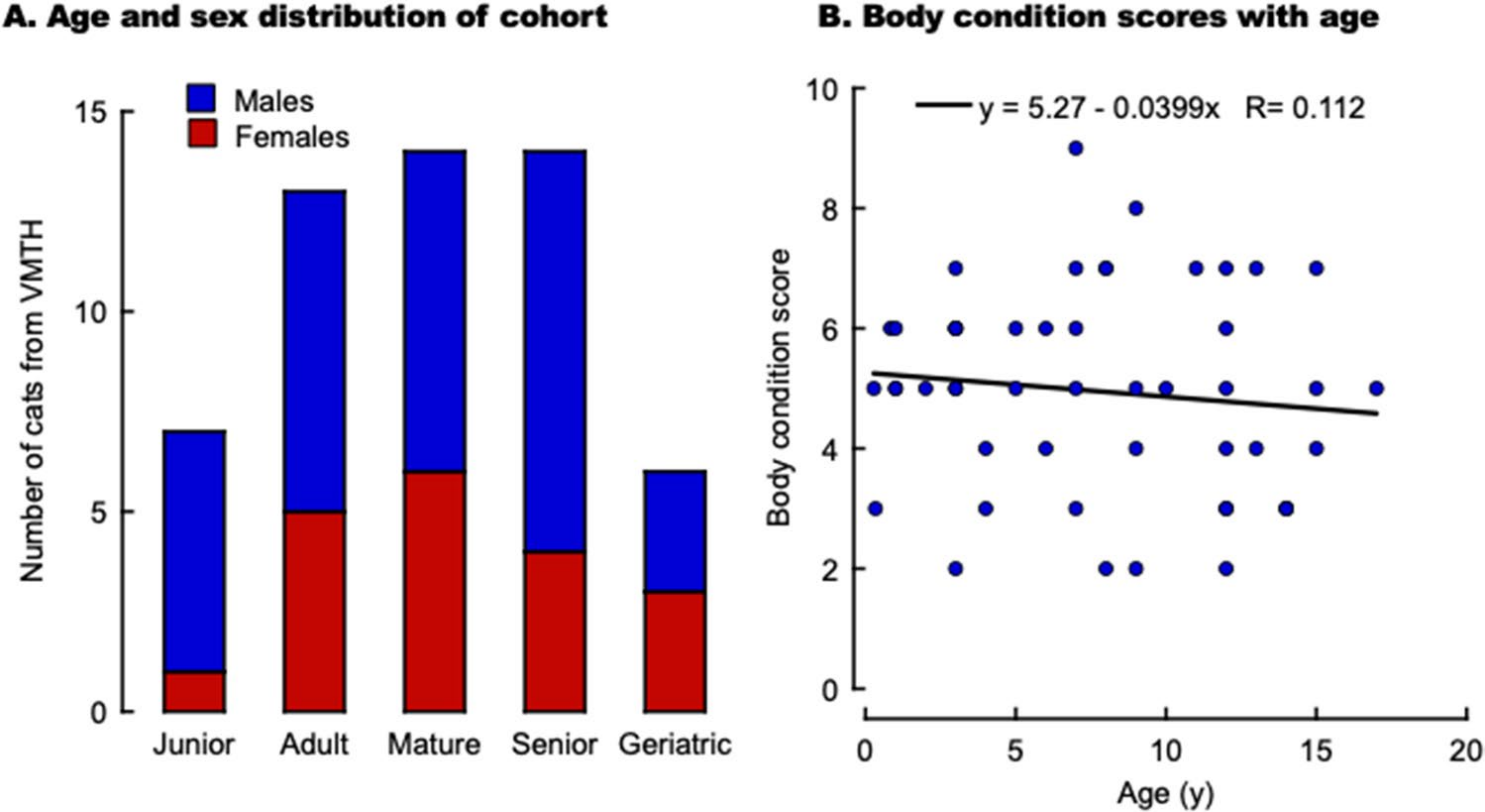
Age, sex, and body score condition distribution in domestic cats from the Veterinary Medicine Teaching Hospital from Dec 2022–Jan 2023. (**A**) Age and sex distribution of a subset of 54 domestic cats from all those seen at the VMTH between Dec 2022 and Jan 2023. (**B**) Association between body condition scores and cat's age. The equation represents the linear fitting.

Blood samples were collected from 54 male (n = 35) and female (n = 19) cats that visited the VMTH for a medical consultation between December 2022 and January 2023, from which blood samples were available ranging from 3 months to 17 y old of age (Fig. 3A; Table 2). This cohort had more males (male-to-female ratio = 1.8 from the VMTH vs. 0.15 from the Cat Colony) and a more comprehensive representation of ages than those at the Cat Colony: junior (1–2 y; n = 7), adults (3 to 6 y; n = 13), mature cats (7–10 y; n = 14), seniors (11–14 y; n = 14) and geriatric cats (≥ 15 y; n = 6). Significantly different from the cohort from the Cat Colony, most animals were not sexually intact (all males were neutered except one, and all females were spayed). The most common diagnosis was chronic kidney disease, followed by lymphoma, heart disease, and hypocobalaminemia (Table 2). The diet of most cats was unknown, but for those with information on dietary habits, most were fed commercially available brands such as Purina, Royal Canin, and Costco (Table 2). Similar to the Cat Colony data (Fig. 1B), the VMTH cohort did not show a statistically significant correlation between body condition scores and age (Fig. 3B).

**Figure 4:**
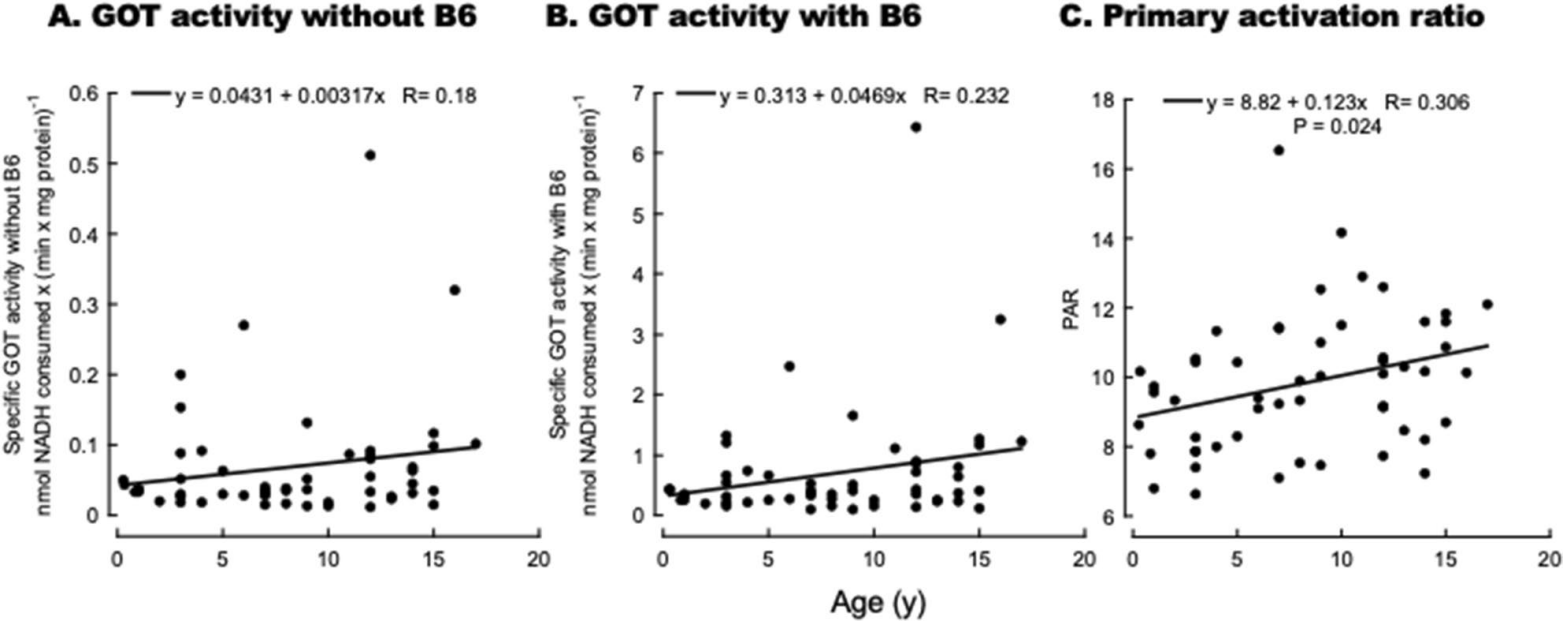
Glutamate–oxaloacetate transaminase specific activity with and without vitamin B6 addition as a function of age. GOT activity was evaluated in hemolysates as described in detail under “Materials and methods” without (**A**) and with (**B**) PLP addition. The data are presented as a function of the cat's age. Equations represent the linear fittings, and the P values are from Pearson's. The primary activation ratio (ratio of the GOT-specific activity with and without PLP addition) is shown as a function of age (**C**).

As described before, the specific GOT activity was assessed in whole blood samples with and without the addition of saturating PLP concentrations. The GOT activities without or with PLP addition did not show a statistically significant correlation with age (Fig. 4A,B). The differences in the y-axis intercept of these plots compared to those obtained with the Cat Colony cohorts reflect the utilization of isolated red blood cells (Cat Colony) vs. whole blood hemolysates (VMTH cohort). Indeed, the ratio of the y-intercepts was in average 2.25 Cat Colony/VMTH cohorts, consistent with the volume of red blood cells to total blood.

The PAR with the VMTH cohort showed a positive and statistical correlation (Fig. 4C), consistent with the results obtained before with the Cat Colony cohort (Fig. 2C). When the analysis was run separated by sexes, the correlations and slopes were similar (n males = 35, PAR = 9.03 + 0.126 * Age with r^2^ = 0.313; P = 0.067; n females = 19, PAR = 8.42 + 0.119 * Age, r^2^ = 0.313, P = 0.192) with a higher significance for males likely due to the larger number of animals. Indeed, a power analysis indicated that at a fixed alpha of 0.05, the power for females was 0.249, whereas that for males was 0.451. These results were not consistent with those of humans, showing a gender effect for PLP levels^43^.

As described in the “Materials and methods”, the PAR values were corrected for age by using the equation obtained with the healthy Cat Colony cohort. Under this condition, 11 cats (2 spayed females, 8 neutered males, and one sexually intact male) were identified as having abnormally high PAR values normalized to age. This B6 deficient population represented the 20.3% of those tested from the VMTH cohort, as expected, a value lower than that of the Cat Colony cohort (12.8%) but without reaching statistical significance (P = 0.317; Chi-squared test). The age distribution of these 11 cats included 7 juniors (0.25 y, 0.33 y, 0.83 y, three 1-y-old, 2 y), three adults (two 3 y-old, 4 y), and one mature (7 y). In terms of BCS, most cats had abnormal BCS (64%; 3 underweight and 4 overweight).

### Contributors to vitamin B6 status in cats

**Figure 5:**
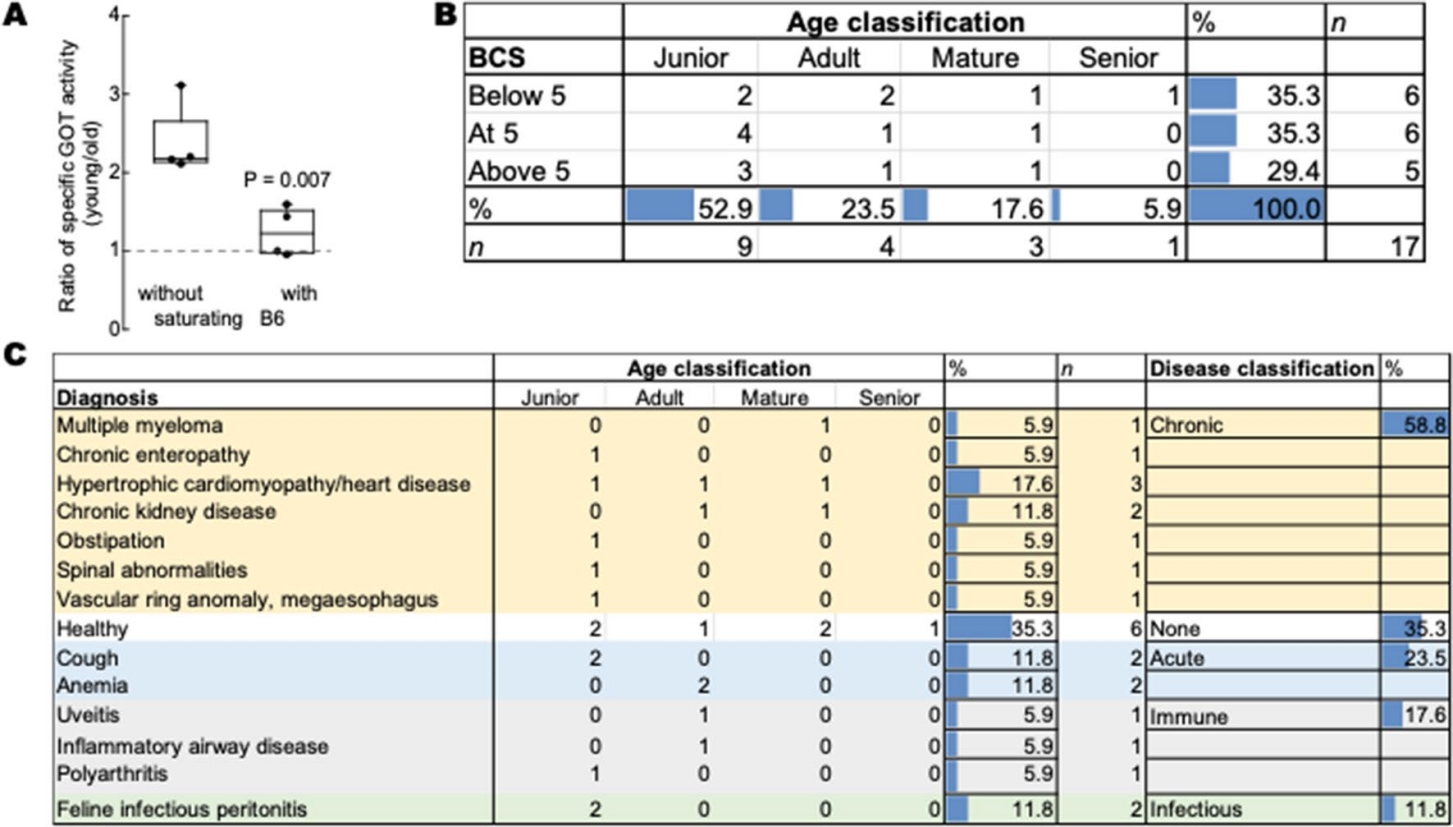
Characterization of the cats identified as B6-deficient. (**A**) Differential activation by PLP of GOT from samples obtained from junior (≤ 2 y old) and old (adults, mature, seniors, and geriatric) cats from the Cat Colony and VMTH (n = 101). Data were presented as the ratio of the specific GOT activity (average junior/average adult, average junior/ average senior, average junior/average/senior, and average junior/average geriatric) with and without PLP addition. P-value was obtained from Student's t-test with equal variance. (**B**) Classification of the 17 cats (11 from VMTH and 6 from the Cat Colony) identified as vitamin B6 deficient based on age and body condition score (BCS). (**C**) Diseases or conditions diagnosed in the 17 cats identified as vitamin B6 deficient. If a cat was diagnosed with more than one condition, all were included as separate entries.

Higher PAR effect may reflect not only poorer vitamin B6 status, but also lower plasma phosphatase activity (activity required to release pyridoxal from PLP for tissue uptake), and lower hepatic albumin secretion (needed for PLP transport) as suggested for humans^72–75^. To ascertain whether PLP or other factors were contributors to this effect, we recalculated the data from the Cat Colony and the VMTH (*n* = 101) in terms of a specific activity ratio (i.e., average specific GOT activity from junior divided by the average specific GOT activity from each age group, namely adults, mature, seniors, and geriatric). The initial specific activity ratio without PLP addition decreased with the addition of PLP (from 2.4 ± 0.2 to 1.2 ± 0.2; Fig. 5A). As the GOT activity from older animals with PLP addition was similar to that of younger animals (ratio close to 1), it was concluded that PLP addition accounts for most differences in enzymatic activities between younger and older animals, precluding a significant role for additional factors (83.3% for PLP, 16.6% to other factors). This result is similar to that reported for humans in which PLP status had a major contribution (77%). However, as in humans, the role of factors other than B6 that accounted for the residual difference in GOT activities cannot be excluded (for humans 23%^75^).

To explore other putative contributors to the vitamin B6 status in domestic cats, we gathered more information on the 17 cats identified as vitamin B6 deficient (6 from the Cat Colony and 11 from the VMTH) as judged by the age-normalized PAR threshold. The sex distribution in those cats identified as B6 deficient was almost equal (41.2% females and the rest males). These percentages aligned to the distributions already noted in each of the cohorts tested. Based on the body condition scores (Fig. 5B), B6-deficient cats were distributed almost equally across the three categories of BCS (below 5, at 5 or above 5). In terms of age, most cats with vitamin B6 deficiency were the junior-adult age range (9 and 4 or 13 of 17; Fig. 2B). For older cats, those with BCS ranging from underweight to normal were linked to vitamin B6 deficiency.

Changes in vitamin B6 status have been linked to numerous human diseases and conditions. For instance, GOT apoenzyme contents increase in diseases related to necrotic processes, while decreases had been reported following alcohol intake^48,61^. Low plasma PLP in older human adults is not explained by low dietary vitamin B6 or low protein intake^24,76^, deficit in absorption, impaired synthesis or retention of PLP in erythrocytes or liver^77^. However, some^78^ but not all^77,79^ studies suggest increased catabolism related or not to age-dependent decreases in albumin and, in particular, with an increase in alkaline phosphatase^64,77,80^. At the same time, the rise in plasma PA (which may be taken wrongly as increased catabolism) in both older men and women may partly be explained by impaired renal function^80^. The data from the 17 vitamin B6-deficient cats were analyzed in terms of medical diagnosis (Fig. 5C). As the diagnoses were varied, we used the clustering and classification of the disease’s paradigm developed by Webster et al*.*^81^. This analysis indicated that most cats had a chronic disease (58.8%), followed by acute (23.5%), immune (17.6%) and infectious diseases (11.8%).

**Figure 6:**
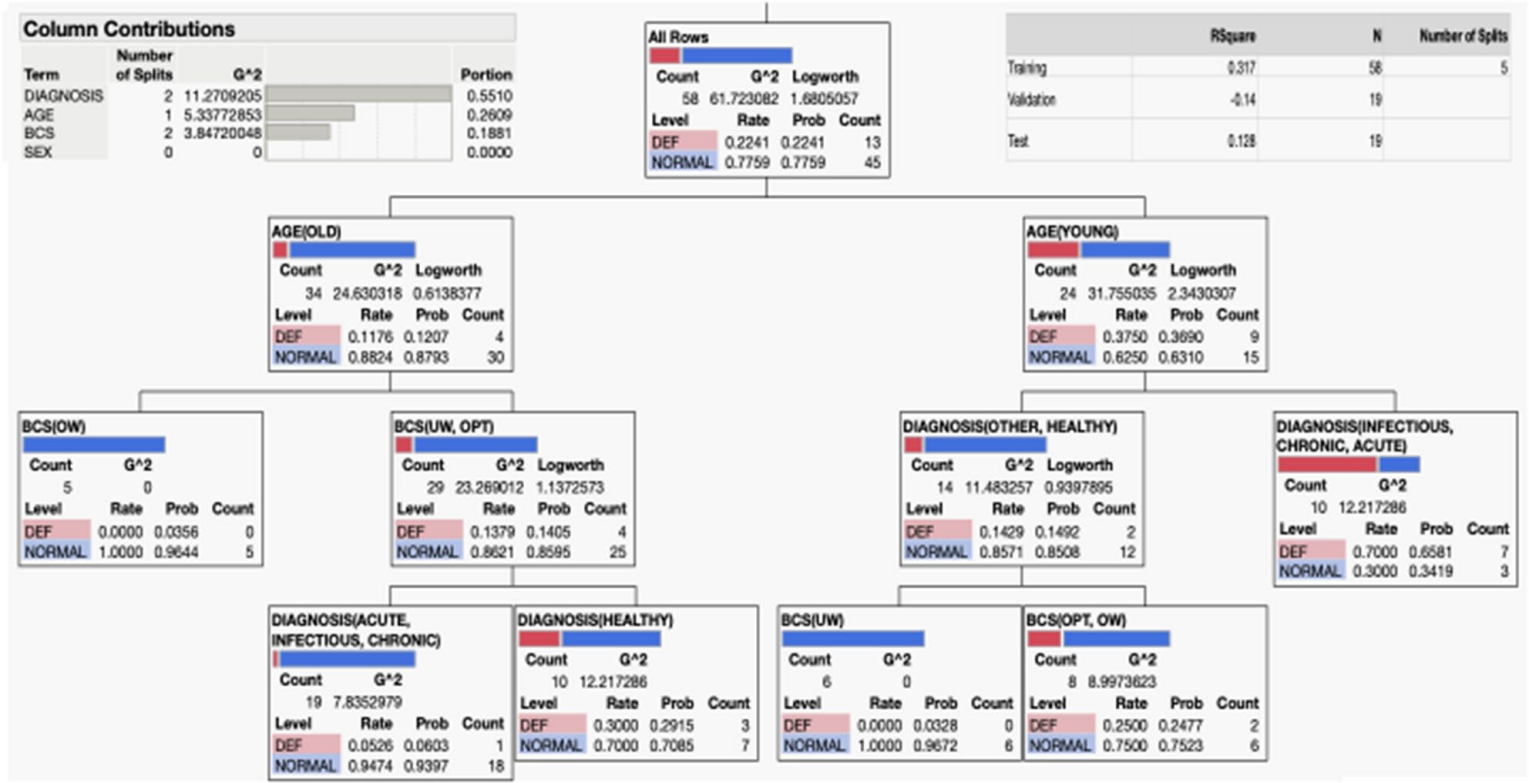
Decision tree of vitamin B6 deficiency in cats. A decision or partition tree was built by utilizing data from 101 cats. The variables utilized were vitamin B6 status (deficient or DEF and normal), age (either as young or old; young, included junior and adult whereas old included the rest), BCS (optimal or OPT, underweight or UW or overweight OW), diagnosis (healthy, chronic, acute, immune, infectious, other), and sex (female or male regardless of their neutered or spayed status). The feature best split was used which maximized splitting and pruning of the tree branches to prevent overfitting. Complete report is found under the Supplementary information.

While the analysis of the 17 cats identified as B6-deficient was informative, we turned to use machine learning to build a partition-based model (or decision Tree) to identify the most important factors that predict vitamin B6 deficiency in all 101 cats and use the resulting tree to make predictions for new observations. Essentially a decision tree is a type of supervised machine learning used to categorize or make predictions based on how a previous set of questions were answered. In our case, we used a partition of 60%, 20% and 20% for training, validation (made with stratified sampling) and testing (Fig. 6; Supplementary information). The model is a form of supervised learning, meaning that the model is trained and tested on a set of data that contains the desired categorization (in our case, vitamin B6 deficiency). The model resulted in 5 splits, in which young (junior to adults) with a diagnosis of infectious, chronic or acute were more likely to be vitamin B6 deficient. Within the young and healthy ones, those with body weights from normal to overweight seemed to be more likely to be vitamin B6 deficient. In older animals (mature, senior and geriatric), underweight to normal body weight seemed to play a more prominent role (Fig. 6). In terms of contributions, the main factor influencing vitamin B6 deficiency was the diagnosis of an infectious, chronic or acute condition, followed by age, and BCS. Sex, in contrast to humans, did not seem to be significant with the cohort analyzed.

## Concluding remarks

In this study, and for the first time, a functional biomarker of vitamin B6 status has been obtained from domestic cats and established a clear path to define vitamin B6 deficiency. The PAR declined with age, and by normalizing these values to age, we established a cut-off value that could be used to identify vitamin B6 deficiency. We also identified key contributors to vitamin B6 deficiency for the first time: disease, followed by age and BCS. Current regulatory minimum for B6 in cat foods is 4 mg/kg dry matter or 1 mg/1000 kcal. These amounts are double the NRC minimum requirement for adult maintenance. However, we would like to stress that these values are for healthy, adult cats. As indicated before, for growing, healthy kittens, 1–2 mg and > 2 mg of B6 seemed required for diets with 30% or 60% protein, respectively^40^. Since our study showed that diagnosis of an infectious, chronic, or acute condition, age, and body condition score seem to affect B6 status in cats, the normalized PAR values may estimate the range of B6 supplementation needed. Given that the average PAR values for those cats deficient in B6 was 1.5 ± 0.2 (n = 17; mean ± SD), then assuming that the PAR is proportional to the deficiency, it would not be unreasonable to suggest a 50% supplementation of B6 in the form of B-complex, or B6 given separately, in junior-adult cats with any of the above mentioned medical conditions or healthy ones with body weights from normal to overweight. Similar to our results, in humans, various chronic diseases were associated with low levels of plasma PLP^82^, including rheumatoid arthritis, inflammatory bowel disease, cardiovascular disease, deep vein thrombosis, diabetes, and cancer^1,14,15,83–90^. An inverse relationship was found between the inflammatory marker C-reactive protein and plasma PLP status^91,92^, the acute-phase protein alpha_1_-acid glycoprotein, tumor necrosis factor-α, and the proinflammatory cytokine interleukin-6 in rheumatoid arthritis and rheumatoid arthritis and inflammatory bowel disease^15,87,93^.

In older cats, body weights from normal to underweight seem to be linked to B6 deficiency. Our data suggested that low food intake and/or age-linked malassimilation might play a role in B6 deficiency status based on the increased energy requirements and compromised digestibility in older cats^94–96^ which is linked to a higher proportion of underweight elderly cats with lower body condition scores^97^. In this regard, the cats from the Cat Colony are fed a diet with appropriate nutrient concentrations (Purina Cat Chow Complete Formula), and no indication of dietary issues was recorded in the VMTH records for the cohort of cats tested.

Our study supports that vitamin B6 dietary supplementation may be indicated in junior to adult animals diagnosed with an infectious, chronic, or acute condition or healthy cats with body weight ranging from optimal to overweight. In older cats, even if healthy, underweight to optimal cats appear to be at risk of vitamin B6 deficiency.

It is important to note the study's limitations: while this report analyzed the largest feline population for vitamin B6 deficiency, the many different medical diagnoses of those from the VMTH made it difficult to identify a specific one. In addition, some of the medical records had limited information in terms of the detailed history of the animals. The content of B6 in diets for those animals at the VMTH was hampered by the proprietary nature of some of the formulations. We also cannot exclude the possible effect of differences in neuter/spay status and sex between the Cat Colony and VMTH. The former had mainly females, none were neutered/spayed, and all were from either a single colony (Cat Colony) or single hospital (VMTH). While a functional biomarker of B6 was informative in identifying B6 deficiency, further research, ideally prospective in nature, would need to be performed to identify the efficacy of specific vitamin B6 supplementation regimens. Finally, further studies are needed to thoroughly characterize both positive and negative clinical effects of B6 supplementation.

## Materials and methods

### Ethics statement

The ethics of this study was approved by the IACUC (protocol number 21780). All experimental procedures were strictly performed per the ethical requirements of IACUC at the University of California Davis.

### ARRIVE guidelines

This study was fully aligned with the ARRIVE 2.0 guidelines^98^, ensuring transparent and comprehensive reporting of the research methods utilized in this study and its outcomes. Adhering to these guidelines enhances research quality and reproducibility, underscoring our commitment to upholding rigorous standards and promoting transparency in our findings. We confirm that all experiments were performed in accordance with relevant guidelines and regulations.

### Animal subjects

The experimental protocol adhered to the Guide for the Care and Use of Laboratory Animals (NRC 1985) and was approved by the Institutional Animal Care and Use Committee (IACUC) of the University of California-Davis. The study population included 60 sexually intact, specific-pathogen-free domestic shorthair cats (*Felis catus*) aged 1 to 17 y from the Feline Nutrition and Pet Care Center School of Veterinary Medicine, the University of California-Davis (Table 1). Pathogens tested in this cohort included feline calici, herpes, leukemia, and immunodeficiency viruses (all negative). All animals present in the colony between March and July 2021 were included in this study, and these criteria were established a priori. All cats underwent a veterinary examination and were determined to be healthy and able to participate in the study as judged by the standing veterinarians in the facility.

Cats in this facility are housed in large group enclosures with enrichment. Cats were group-housed in large wire cages (2.5 × 2.5 × 2.5 m) in humidity- and temperature-controlled rooms (21 ± 2 °C) with a light: dark cycle of 14 h:10 h. The cats had habitual free access to tap water and a commercially available, balanced, dry expanded diet formulated for all life stages (Purina Cat Chow Complete Formula, Nestle Purina PetCare Company, St. Louis, MO; minimum 1 mg pyridoxine/1000 kcal^99^). Staff veterinarians evaluated the body condition score with a scale from 1 to 9 and a score of 5 as optimal^66^.

The other study population included 54 cats aged 3 months to 17 years old brought to the Veterinary Medical Teaching Hospital at the University of California, Davis, between December 2022 and January 2023 to seek care under different circumstances (Table 2). At the hospital, each cat was assessed by various clinicians, technical staff, and veterinary students who took the consultation (assigned by availability and expertise). Thus, all information on the chart of each cat was done by different clinicians specialized in small animal medicine, independently of those at the Cat Colony.

### Sample collection

Blood collection across subjects from the Cat Colony was done randomly, and the number of subjects bled/day varied depending on the technician's schedule and workload. Blood collection was adequately performed for most cats while the animal was awake using the appropriate restraint. Restraint was necessary to prevent movement resulting in blood vessel or other organ lacerations and serious complications. Only trained staff performed this procedure by following the criteria to determine the maximum, safe amount of blood to withdraw. The approximate single blood draw was < 5 ml for a 5 kg cat, following the University's IACUC guidelines. This volume represents an estimate depending on the animal's size, health, and hydration status. Cats were not returned to their enclosure until complete hemostasis (i.e., no more blood coming from the collection site as determined using gauze and direct pressure). Up to several minutes of pressure sometimes was required following jugular vein puncture. Collecting blood from the medial saphenous, cephalic, and jugular is recommended for cats. We chose to use the latter site because it does not require anesthesia, and the trained staff have ample experience with this procedure.

Blood samples collected at the VMTH followed standard and approved protocols.

All blood samples from both populations were collected using disposable syringes and needles; samples were transferred into 2‐ml tubes containing K_2_EDTA (Sarstedt, Nümbrecht, Germany). For measurement of enzymatic activities and protein concentrations, 6 ml of blood was aliquoted into 3 EDTA tubes (2 mL/tube). Blood samples were stored at 4 °C until analysis and were analyzed 7–8 h after phlebotomy. Blood samples were collected in the morning to avoid any putative diurnal cycles. After centrifugation at 3000×*g* at 4 °C, plasma was removed, and the equivalent 1 ml of packed red blood cells was washed in phosphate-buffered saline three times and suspended in 1 ml of a 1% solution of Triton X‐100 double density, peroxide‐free detergent (Sigma, St. Louis, MO, USA). If needed, washed and permeabilized red cells were stored at − 30 °C for a maximum of 3 months or at − 70 °C for up to 18 months. After homogenization and incubation at 25 °C for 10‐min, the hemolysate was centrifuged at 13,000*g* for 10 min at 4 °C.

### GOT activity assessment

Blood hemolysates (10 µl) were homogenized and diluted 1/100 with 50 mM phosphate buffer (pH 7.5) containing 0.2% bovine albumin and 2 mM K_2_EDTA. 1/20 with phosphate saline buffer. Each sample was run in duplicates. If values exceeded a coefficient of variation higher than 10%, they were re-assessed. Each sample was tested without and with 20 µM PLP addition. For each of these conditions, a blank and a complete system were run (for a total of 8 wells/sample) in which the "blank" contained 0.1 M Tris–HCl, 0.5 EDTA, 0.2 mM freshly prepared NADH, 10 µM l-aspartate (pH 8.0), and 10 mU malate dehydrogenase. In contrast, the complete system had 4–5 µl of diluted hemolysates. The decrease in absorbance at 340 nm was followed for 15 min in a Tecan Microplate reader at 37 °C. The blank rates were subtracted from those rates with hemolysates. All reagents were of analytical grade and obtained from Sigma Chemical Company. The assay of this enzyme followed essentially that published by Buetler^100^. The following parameters and ratios were calculated for each blood sample: the GOT activity with and without addition of PLP was expressed in units of enzymatic activity per mg protein (1 U = 1 µmol × min^−1^) and calculated as [ΔAbs 340 nm/min × (6.22 µmol/ml)^−1^] = µmol/min × (ml)^−1^ × (mg of protein × ml^−1^)^−1^ = U/mg. The primary activation ratio (PAR) is the ratio of the activity in the presence of a given PLP concentration (saturating) to that in the absence of added PLP. Protein determination was performed using the bicinchoninic acid method (Pierce™BCA protein assay kit, catalog #23225).

Considering that the PAR values correlated with age, the mean PAR values were corrected for age to result in cut-off values used to identify marginal vitamin B6 deficiency. Since the PAR values normalized to age followed a normal distribution (Supplementary information), 1.65 × SD results in a tail that gives a probability of 5% of the data being excluded from the distribution. If this value is added to the mean, anything above this value has a < 5% probability of being significant.

### Statistical analyses

Enzymatic activities were assessed in technical triplicates. Descriptive statistics were generated for each parameter and analyzed for normality by the Kolmogorov‐Smirnov test. For all analyses, *P* ≤ 0.05 was considered significant. Data from GOT activities and PAR were analyzed by using the Fit Model feature under JMP Pro software (version 17.0.0), selecting stepwise regression and using as covariates age, BCS, and sex or if using only one sex, BCS, and age. The model selection criteria were defined by the minimum Bayesian Information Criterion (BIC) as the stopping rule. Then the model was then run using least squares fit (Supplementary information). The decision tree was run as a partition-based model and utilizing a 60:20:20 for the training, validation (stratified sampling) and testing as indicated under Supplementary information.

### Supplementary Information

Supplementary Information.

## Data Availability

All data generated and analyzed in this study are included in the published article.
