# Supplementary material for: Factors influencing vitamin B6 status in domestic cats: age, disease, and body condition score
Source: Sci Rep. 2024 Jan 23;14:2037. doi: 10.1038/s41598-024-52367-y (PMC10806207; doi:10.1038/s41598-024-52367-y)
Supplement: Supplementary file 1 — Supplementary Information. [file 41598_2024_52367_MOESM1_ESM.pdf]

Response Activity(no B6)

Whole Model

Actual by Predicted Plot

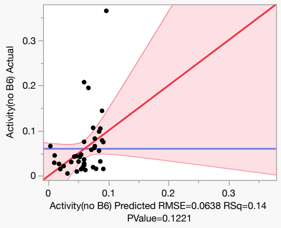

Effect Summary

Source	Logworth	PValue
Age (y)	1.304	0.04961
BCS	0.363	0.43369
Sex	0.168	0.67908

Lack Of Fit

Source	DF	Sum of Squares	Mean Square	F Ratio
Lack Of Fit	27	0.12095805	0.004480	1.4514
Pure Error	11	0.03395279	0.003087	Prob > F
Total Error	38	0.15491083		0.2629
				Max RSq
				0.8114

Residual by Predicted Plot

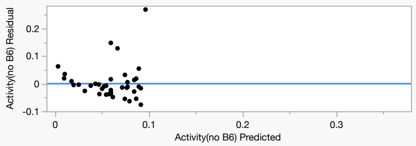

Summary of Fit

RSquare	0.139725
RSquare Adj	0.071809
Root Mean Square Error	0.063848
Mean of Response	0.060088
Observations (or Sum Wgts)	42

Analysis of Variance

Source	DF	Sum of Squares	Mean Square	F Ratio
Model	3	0.02516047	0.008387	2.0573
Error	38	0.15491083	0.004077	Prob > F
C. Total	41	0.18007130		0.1221

Parameter Estimates

Term	Estimate	Std Error	t Ratio	Prob>|t|
Intercept	0.1240876	0.044228	2.81	0.0079*
Age (y)	-0.004976	0.002453	-2.03	0.0496*
Sex[F]	0.0059937	0.014376	0.42	0.6791
BCS	-0.007133	0.009015	-0.79	0.4337

Effect Tests

Source	Nparm	DF	Sum of Squares	F Ratio	Prob > F
Age (y)	1	1	0.01676665	4.1129	0.0496*
Sex	1	1	0.00070862	0.1738	0.6791
BCS	1	1	0.00255252	0.6261	0.4337

Age (y)

Leverage Plot

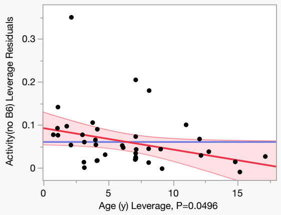

Sex

Leverage Plot

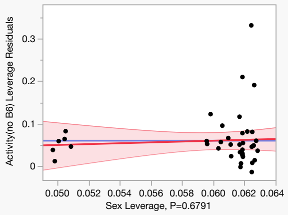

Least Squares Means Table

Level	Least Sq Mean	Std Error	Mean
F	0.06180024	0.01067394	0.060884
M	0.04981275	0.02654099	0.055311

BCS

Leverage Plot

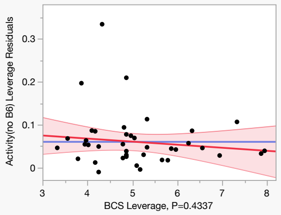

GOT activity without PLP supplementation from the Cat Colony cohort, including both sexes, age, and BCS.

Response Activity(no B6)

Whole Model

Actual by Predicted Plot

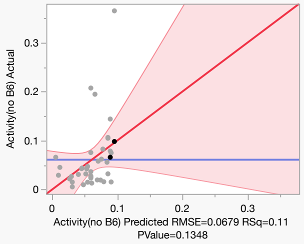

Effect Summary

Source	Logworth	PValue
Age (y)	0.961	0.10938
BCS	0.282	0.52266

Lack Of Fit

Source	DF	Sum of Squares	Mean Square	F Ratio
Lack Of Fit	22	0.11805686	0.005366	1.7385
Pure Error	11	0.03395279	0.003087	**Prob > F**
Total Error	33	0.15200965		0.1716
				**Max RSq**
				0.8022

Residual by Predicted Plot

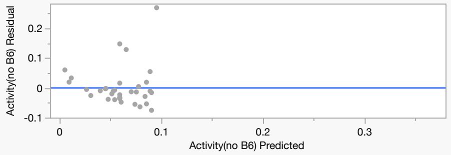

Summary of Fit

RSquare	0.114379
RSquare Adj	0.060705
Root Mean Square Error	0.06787
Mean of Response	0.060884
Observations (or Sum Wgts)	36

Analysis of Variance

Source	DF	Sum of Squares	Mean Square	F Ratio
Model	2	0.01963231	0.009816	2.1310
Error	33	0.15200965	0.004606	**Prob > F**
C. Total	35	0.17164196		0.1348

Parameter Estimates

Term	Estimate	Std Error	t Ratio	Prob>|t|
Intercept	0.1261213	0.049578	2.54	0.0158*
Age (y)	-0.004975	0.003024	-1.65	0.1094
BCS	-0.006368	0.009855	-0.65	0.5227

Effect Tests

Source	Nparm	DF	Sum of Squares	F Ratio	Prob > F
Age (y)	1	1	0.01247073	2.7073	0.1094
BCS	1	1	0.00192308	0.4175	0.5227

Age (y)

Leverage Plot

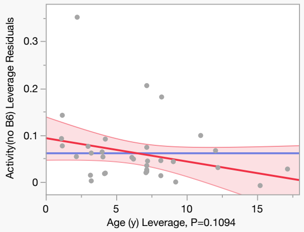

BCS

Leverage Plot

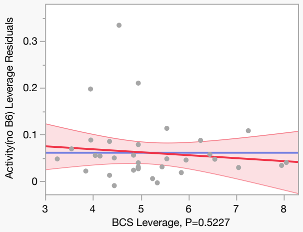

GOT activity without PLP supplementation from the Cat Colony cohort, females only, age, and BCS.

Response Activity(with B6)

Whole Model

Actual by Predicted Plot

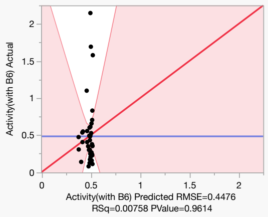

Effect Summary

Source	Logworth	PValue
Sex	0.206	0.62275
Age (y)	0.071	0.84898
BCS	0.027	0.93974

Lack Of Fit

Source	DF	Sum of Squares	Mean Square	F Ratio
Lack Of Fit	27	4.4421764	0.164525	0.5708
Pure Error	11	3.1705241	0.288229	Prob > F
Total Error	38	7.6127005		0.8853
				Max RSq
				0.5867

Residual by Predicted Plot

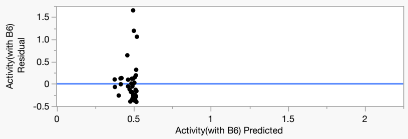

Summary of Fit

RSquare	0.007577
RSquare Adj	-0.07077
Root Mean Square Error	0.447587
Mean of Response	0.482219
Observations (or Sum Wgts)	42

Analysis of Variance

Source	DF	Sum of Squares	Mean Square	F Ratio
Model	3	0.0581211	0.019374	0.0967
Error	38	7.6127005	0.200334	Prob > F
C. Total	41	7.6708216		0.9614

Parameter Estimates

Term	Estimate	Std Error	t Ratio	Prob>|t|
Intercept	0.4921799	0.310045	1.59	0.1207
Age (y)	-0.003297	0.017199	-0.19	0.8490
Sex[F]	0.0499862	0.100779	0.50	0.6228
BCS	-0.004809	0.063196	-0.08	0.9397

Effect Tests

Source	Nparm	DF	Sum of Squares	F Ratio	Prob > F
Age (y)	1	1	0.00736336	0.0368	0.8490
Sex	1	1	0.04928524	0.2460	0.6228
BCS	1	1	0.00116020	0.0058	0.9397

Age (y)

Leverage Plot

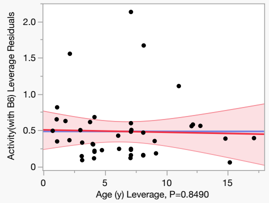

Sex

Leverage Plot

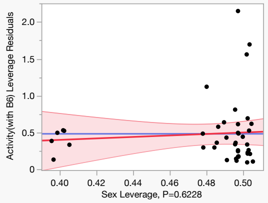

Least Squares Means Table

Level	Least Sq Mean	Std Error	Mean
F	0.49650067	0.07482616	0.495886
M	0.39652830	0.18605682	0.400219

BCS

Leverage Plot

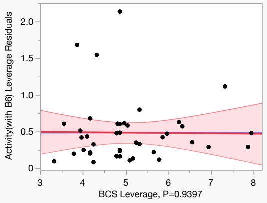

GOT activity with PLP supplementation from the Cat Colony cohort, including both sexes, BCS, and age.

Response Activity(with B6)

Whole Model

Actual by Predicted Plot

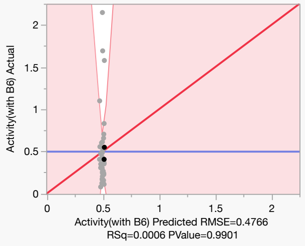

Effect Summary

Source	Logworth	PValue
Age (y)	0.043	0.90612
BCS	0.012	0.97386

Lack Of Fit

Source	DF	Sum of Squares	Mean Square	F Ratio
Lack Of Fit	22	4.3257854	0.196627	0.6822
Pure Error	11	3.1705241	0.288229	**Prob > F**
Total Error	33	7.4963095		0.7860
				**Max RSq**
				0.5773

Residual by Predicted Plot

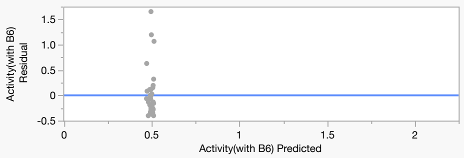

Summary of Fit

RSquare	0.000601
RSquare Adj	-0.05997
Root Mean Square Error	0.476614
Mean of Response	0.495886
Observations (or Sum Wgts)	36

Analysis of Variance

Source	DF	Sum of Squares	Mean Square	F Ratio
Model	2	0.0045077	0.002254	0.0099
Error	33	7.4963095	0.227161	**Prob > F**
C. Total	35	7.5008172		0.9901

Parameter Estimates

Term	Estimate	Std Error	t Ratio	Prob>|t|
Intercept	0.524094	0.348156	1.51	0.1417
Age (y)	-0.002523	0.021234	-0.12	0.9061
BCS	-0.002285	0.069206	-0.03	0.9739

Effect Tests

Source	Nparm	DF	Sum of Squares	F Ratio	Prob > F
Age (y)	1	1	0.00320837	0.0141	0.9061
BCS	1	1	0.00024764	0.0011	0.9739

Age (y)

Leverage Plot

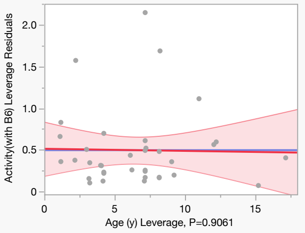

BCS

Leverage Plot

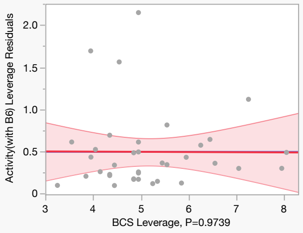

GOT activity with PLP supplementation from the Cat Colony cohort, females only, age, and BCS.

Response PAR

Whole Model

Actual by Predicted Plot

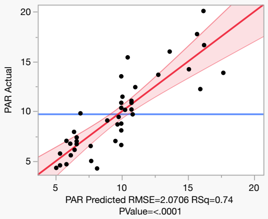

Effect Summary

Source	Logworth	PValue
Age (y)	11.122	0.00000
Sex	0.611	0.24469
BCS	0.479	0.33155

Lack Of Fit

Source	DF	Sum of Squares	Mean Square	F Ratio
Lack Of Fit	27	129.45720	4.79471	1.5762
Pure Error	11	33.46164	3.04197	Prob > F
Total Error	38	162.91884		0.2162
				Max RSq
				0.9473

Residual by Predicted Plot

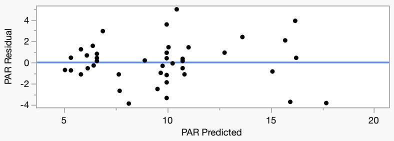

Summary of Fit

RSquare	0.743237
RSquare Adj	0.722966
Root Mean Square Error	2.070589
Mean of Response	9.709094
Observations (or Sum Wgts)	42

Analysis of Variance

Source	DF	Sum of Squares	Mean Square	F Ratio
Model	3	471.59190	157.197	36.6655
Error	38	162.91884	4.287	Prob > F
C. Total	41	634.51073		<.0001*

Parameter Estimates

Term	Estimate	Std Error	t Ratio	Prob>|t|
Intercept	3.6560742	1.434304	2.55	0.0150*
Age (y)	0.7730835	0.079564	9.72	<.0001*
Sex[F]	-0.550898	0.466214	-1.18	0.2447
BCS	0.2875499	0.292353	0.98	0.3315

Effect Tests

Source	Nparm	DF	Sum of Squares	F Ratio	Prob > F
Age (y)	1	1	404.76987	94.4105	<.0001*
Sex	1	1	5.98630	1.3963	0.2447
BCS	1	1	4.14762	0.9674	0.3315

Age (y)

Leverage Plot

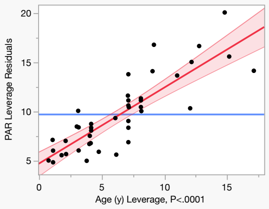

Sex

Leverage Plot

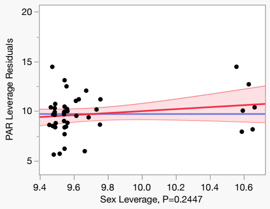

Least Squares Means Table

Level	Least Sq Mean	Std Error	Mean
F	9.551694	0.34615428	9.6159
M	10.653489	0.86071994	10.2683

BCS

Leverage Plot

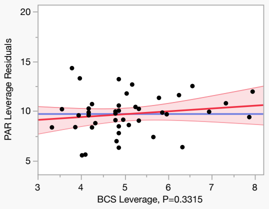

PAR values from the Cat Colony cohort, including both sexes, BCS, and age

Response PAR

Whole Model

Actual by Predicted Plot

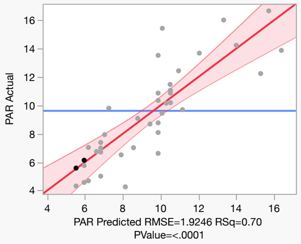

Effect Summary

Source	Logworth	PValue
Age (y)	8.020	0.00000
BCS	0.872	0.13426

Lack Of Fit

Source	DF	Sum of Squares	Mean Square	F Ratio
Lack Of Fit	22	88.76740	4.03488	1.3264
Pure Error	11	33.46164	3.04197	Prob > F
Total Error	33	122.22904		0.3210
				Max RSq
				0.9168

Residual by Predicted Plot

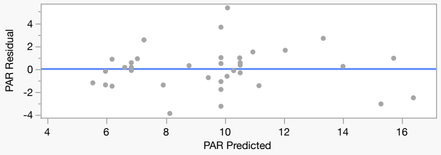

Summary of Fit

RSquare	0.696257
RSquare Adj	0.677848
Root Mean Square Error	1.924555
Mean of Response	9.615894
Observations (or Sum Wgts)	36

Analysis of Variance

Source	DF	Sum of Squares	Mean Square	F Ratio
Model	2	280.18045	140.090	37.8223
Error	33	122.22904	3.704	Prob > F
C. Total	35	402.40949		<.0001*

Parameter Estimates

Term	Estimate	Std Error	t Ratio	Prob>|t|
Intercept	3.163256	1.405843	2.25	0.0312*
Age (y)	0.6516969	0.085741	7.60	<.0001*
BCS	0.4290208	0.27945	1.54	0.1343

Effect Tests

Source	Nparm	DF	Sum of Squares	F Ratio	Prob > F
Age (y)	1	1	213.98113	57.7717	<.0001*
BCS	1	1	8.72988	2.3569	0.1343

Age (y)

Leverage Plot

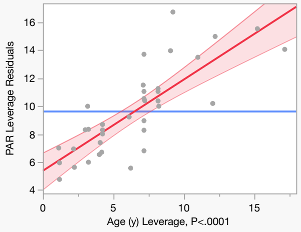

BCS

Leverage Plot

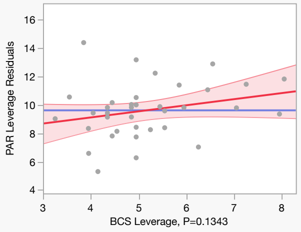

PAR values from the Cat Colony cohort, females only, BCS, and age

Response PAR

Whole Model

Regression Plot

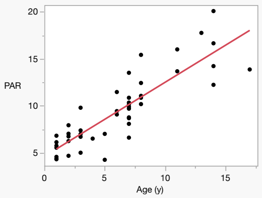

Actual by Predicted Plot

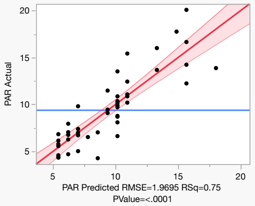

Lack Of Fit

Source	DF	Sum of Squares	Mean Square	F Ratio
Lack Of Fit	10	59.14621	5.91462	1.7936
Pure Error	35	115.41442	3.29755	**Prob > F**
Total Error	45	174.56063		0.0986
				**Max RSq**
				0.8340

Residual by Predicted Plot

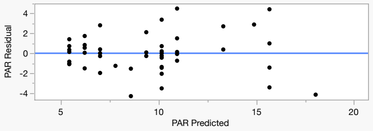

Summary of Fit

RSquare	0.748983
RSquare Adj	0.743405
Root Mean Square Error	1.969549
Mean of Response	9.37613
Observations (or Sum Wgts)	47

Analysis of Variance

Source	DF	Sum of Squares	Mean Square	F Ratio
Model	1	520.85206	520.852	134.2705
Error	45	174.56063	3.879	**Prob > F**
C. Total	46	695.41269		<.0001*

Parameter Estimates

Term	Estimate	Std Error	t Ratio	Prob>|t|
Intercept	4.626185	0.500568	9.24	<.0001*
Age (y)	0.7888601	0.068078	11.59	<.0001*

Effect Tests

Source	Nparm	DF	Sum of Squares	F Ratio	Prob > F
Age (y)	1	1	520.85206	134.2705	<.0001*

Prediction Expression

$$4.626184999 + 0.7888600902 \cdot \text{Age (y)}$$

Age (y)

Leverage Plot

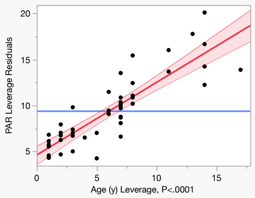

Correlation of PAR values from the Cat Colony cohort with age

Explore Outliers

Commands

Quantile Range Outliers

Outliers are values Q times the interquantile range past the lower and upper quar

Tail Quantile 0.1 | Rescan

Q 3 | Restrict search to integers | Close

Outliers by Column

Show only columns with outliers

Select columns and choose an action.

Identify Outliers in Table: Select Rows | Color Cells | Exclude Rows | Color Rows

Clear Outliers in Table: Add to Missing Value Codes | F | Change to Missing

Column	10% Quantile	90% Quantile	Low Threshold	High Threshold	Number of Outliers	Outliers (Count)
Column 1	0.76614	1.27932	-0.7734	2.81885		0

Explore Outliers

Commands

Robust Fit Outliers

Outliers are K spreads from the center.

- Huber
- Cauchy
- Quartile

K Sigma 4

Rescan

Close

Outliers by Column

Show only columns with outliers

Select columns and choose an action.

Identify Outliers in Table: Select Rows | Color Cells | Exclude Rows | Color Rows

Clear Outliers in Table: Add to Missing Value Codes | Formula Colum | Change to Missing | Formula Scrip

Column	Huber Center	Huber Spread	Huber N Outliers	Cauchy Center	Cauchy Spread	Cauchy N Outliers	Quartile Center
Column 1	1.0005485	0.1994002	0	1.0015303	0.1574966	0	1.0074977

Distributions

normPAR

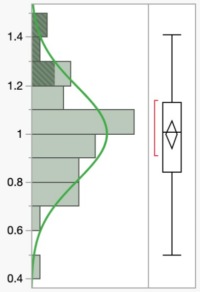

Compare Distributions

Show	Distribution		AICc	BIC	-2*LogLikelihood
☑	Normal		-16.9735	-13.54593	-21.24622

Quantiles

100.0%	maximum	1.4100321
99.5%		1.4100321
97.5%		1.4082337
90.0%		1.2793171
75.0%	quartile	1.1320712
50.0%	median	1.0074977
25.0%	quartile	0.8437466
10.0%		0.7661408
2.5%		0.5278974
0.5%		0.4968816
0.0%	minimum	0.4968816

Summary Statistics

Mean	0.9985885
Std Dev	0.1950842
Std Err Mean	0.028456
Upper 95% Mean	1.0558673
Lower 95% Mean	0.9413096
N	47
N Missing	0

Fitted Normal Distribution

Parameter		Estimate	Std Error	Lower 95%	Upper 95%
Location	μ	0.9985885	0.028456	0.9413096	1.0558673
Dispersion	σ	0.1950842	0.0204504	0.1621101	0.245023

Measures

-2*LogLikelihood	-21.24622
AICc	-16.9735
BIC	-13.54593

Goodness-of-Fit Test

	W	Prob<W
Shapiro-Wilk	0.9852801	0.8118

	A²	Simulated p-Value
Anderson-Darling	0.296814	0.6052

Note: Ho = The data is from the Normal distribution. Small p-values reject Ho.

Distributions

Age (y)

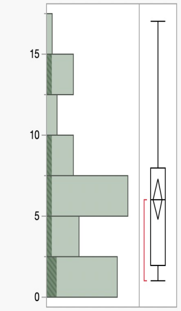

Quantiles

100.0%	maximum	17
99.5%		17
97.5%		16.4
90.0%		14
75.0%	quartile	8
50.0%	median	6
25.0%	quartile	2
10.0%		1
2.5%		1
0.5%		1
0.0%	minimum	1

Summary Statistics

Mean	6.0212766
Std Dev	4.265582
Std Err Mean	0.6221991
Upper 95% Mean	7.2736984
Lower 95% Mean	4.7688548
N	47
N Missing	0

Sex

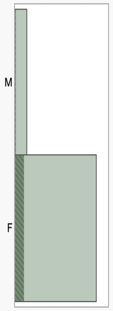

Frequencies

Level	Count	Prob
F	41	0.87234
M	6	0.12766
Total	47	1.00000

N Missing 0

2 Levels

BCS

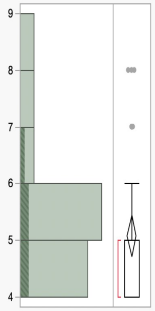

Quantiles

100.0%	maximum	8
99.5%		8
97.5%		8
90.0%		7
75.0%	quartile	5
50.0%	median	5
25.0%	quartile	4
10.0%		4
2.5%		4
0.5%		4
0.0%	minimum	4

Summary Statistics

Mean	5.0714286
Std Dev	1.1768676
Std Err Mean	0.1815946
Upper 95% Mean	5.4381663
Lower 95% Mean	4.7046908
N	42
N Missing	5

normPAR

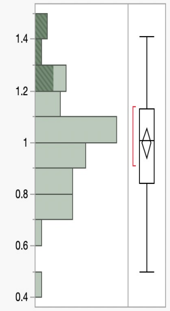

Quantiles

100.0%	maximum	1.4100321
99.5%		1.4100321
97.5%		1.4082337
90.0%		1.2793171
75.0%	quartile	1.1320712
50.0%	median	1.0074977
25.0%	quartile	0.8437466
10.0%		0.7661408
2.5%		0.5278974
0.5%		0.4968816
0.0%	minimum	0.4968816

Summary Statistics

Mean	0.9985885
Std Dev	0.1950842
Std Err Mean	0.028456
Upper 95% Mean	1.0558673
Lower 95% Mean	0.9413096
N	47
N Missing	0

Outlier identification and distribution of normalized PAR values by age from the Cat Colony cohort

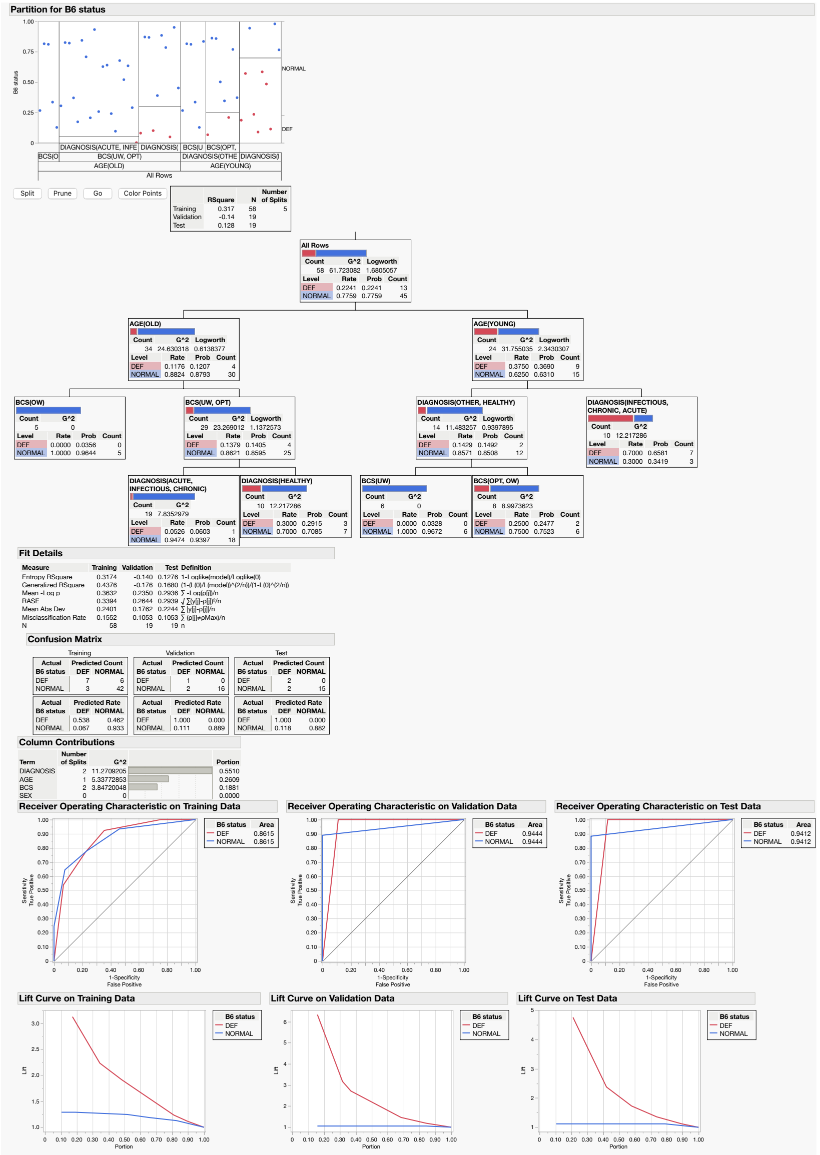

Partition tree statistics was performed with VMTH and Cat Colony cats considering YOUNG (as the sum of juniors and adults) and OLD (mature, senior, and geriatric); BCS classified as optimal (=5), underweight (below 5) and overweight (above 5); diagnosis classified as immune, infectious, acute, chronic, healthy or other); sex (regardless of the spayed/neutered condition) was taken as female or male.
